# Acute Biochemical, Cardiovascular, and Autonomic Response to Hyperbaric (4 atm) Exposure in Healthy Subjects

**DOI:** 10.1155/2018/5913176

**Published:** 2018-05-27

**Authors:** Mariusz Kozakiewicz, Joanna Slomko, Katarzyna Buszko, Wladyslaw Sinkiewicz, Jacek J. Klawe, Malgorzata Tafil-Klawe, Julia L. Newton, Pawel Zalewski

**Affiliations:** ^1^Department of Food Chemistry, Nicolaus Copernicus University in Torun, Ludwik Rydygier Collegium Medicum in Bydgoszcz, Dębowa 3, 85-626 Bydgoszcz, Poland; ^2^Department of Hygiene, Epidemiology and Ergonomics, Nicolaus Copernicus University in Torun, Ludwik Rydygier Collegium Medicum in Bydgoszcz, M. Sklodowskiej-Curie 9, 85-094 Bydgoszcz, Poland; ^3^Department of Theoretical Foundations of Bio-Medical Sciences and Medical Informatics, Nicolaus Copernicus University in Torun, Ludwik Rydygier Collegium Medicum in Bydgoszcz, Jagiellonska 13, 85-067 Bydgoszcz, Poland; ^4^2nd Department of Cardiology, Nicolaus Copernicus University in Torun, Ludwik Rydygier Collegium Medicum in Bydgoszcz, Ujejskiego 75, 85-168 Bydgoszcz, Poland; ^5^Department of Human Physiology, Nicolaus Copernicus University in Torun, Ludwik Rydygier Collegium Medicum in Bydgoszcz, Karłowicza 24, 85-092 Bydgoszcz, Poland; ^6^Institute for Ageing and Health, The Medical School, Newcastle University, Framlington Place, Newcastle-upon-Tyne NE2 4HH, UK

## Abstract

The aim of this study was to explore the effect of a hyperbaric environment alone on the cardiovascular system by ensuring elimination of factors that may mask the effect on hyperbaria. The research was performed in a hyperbaric chamber to eliminate the effect of physical activity and the temperature of the aquatic environment. Biochemical analysis and examination with the Task Force Monitor device were performed before and immediately after exposure. TFM was used for noninvasive examination of the cardiovascular system and the functional evaluation of the autonomic nervous system. Natriuretic peptides were measured as biochemical markers which were involved in the regulation of haemodynamic circulation vasoconstriction (urotensin II). L-arginine acted as a precursor of the level of the nitric oxide whereas angiotensin II and angiotensin (1–7) were involved in cardiac remodeling. The study group is comprised of 18 volunteers who were professional divers of similar age and experience. The results shown in our biochemical studies do not exceed reference ranges but a statistically significant increase indicates the hyperbaric environment is not without impact upon the human body. A decrease in HR, an increase in mBP, dBP, and TPR, and increase in parasympathetic heart nerves activity suggest an increase in heart afterload with a decrease in heart activity within almost one hour after hyperbaric exposure. Results confirm that exposure to a hyperbaric environment has significant impact on the cardiovascular system. This is confirmed both by changes in peptides associated with poorer cardiovascular outcomes, where a significant increase in the studied parameters was observed, and by noninvasive examination.

## 1. Introduction

Diving exposes a body to an unnatural hyperbaric environment which can lead to increased gas dissolved in the tissues [1]. Gas solubility in tissues is a very complex process which cannot be described using Henry's law being a law used for gas solubility in liquids. It is a process for which Dalton's law of partial pressure needs to be taken into consideration and also includes gas solubility in a complex phase system (meaning law of gas solubility in two immiscible liquids). Additional issue is the usage of saturation and dehydration of the tissues which show the speed with which these processes occur and mechanisms of the creation of microbubbles of gas.

The complexity of these processes which take place in the diver's organism during descending and ascending does not allow for its full theoretical description [1, 2].

In a clinical setting, hyperbaric oxygen treatment is used for treatment of decompression sickness, carbon monoxide poisoning, gas gangrene, soft tissue [3–5], postradiation burns, and treatment of poorly healing wounds [6]. Studies have also investigated the use of HBO after radiation or chemotherapy [7, 8], and today there are many clinical indications for the use of HBO therapy [9].

Another form of hyperbaric exposure is the long-term frequent exposure to high external pressure observed among professional divers. This type of diving usually takes place at a significant depths and lasts much longer than recreational diving. When subjected to a hyperbaric environment, divers' bodies experience factors that may influence haemodynamic and cardiovascular (CV) system function [10, 11]. The work of a professional diver is therefore associated with stress related not only to exposure to a hyperbaric environment, but also to heavy physical work. This leads to, among other things, an increased heart rate and pressure variations in the respiratory tract affecting the chest and the venous return [11, 12].

The adaptive mechanisms in the CV system that occur during diving remain unclear [12]. Ongoing studies suggest that diving is associated with changes in oxidative stress and the increased expression of regulatory proteins [13] the consequences of which are unclear. Changes in CV function after recreational or professional diving have been observed in many studies but it has frequently been concluded that confounding factors, such as physical activity or temperature, are the cause of any changes detected [14, 15].

The aim of this study was to explore the effect of a hyperbaric environment alone by ensuring elimination of factors that may mask the effect. The research was performed in a hyperbaric chamber to eliminate the effect of physical activity and the temperature of the aquatic environment. In addition, biochemical analysis was performed on natriuretic peptides (BNP and ANP) involved in the regulation of haemodynamic circulation [16–19] and vasoconstriction (urotensin II) [20]; the level of the endogenic amino acid and the nitrogen oxide precursor (L-arginine) [21–23]; and measurement of parameters involved in cardiac remodeling (angiotensin II and angiotensin (1–7)) [24–28].

All cardiovascular response mechanisms of the cardiovascular system are integrated and regulated by the autonomic nervous system. Thus, the analysis of heart rate and blood pressure variability allows determining the activity and reactivity of autonomic nervous system noninvasively.

## 2. Material and Methods

This study was carried out in accordance with the recommendations of the Bioethics Commission of the Collegium Medicum in Bydgoszcz of Nicolaus Copernicus University in Torun with written informed consent from all subjects. All subjects gave written consent in accordance with the Declaration of Helsinki. The protocol was approved by the Bioethics Commission of the Collegium Medicum in Bydgoszcz of Nicolaus Copernicus University in Torun.

### 2.1. Subjects

The study group is comprised of 18 men (volunteers that agreed to take part in the research) with a mean age of 31.1 ± 5.9 years, working on average for 8.7 ± 4.3 years as professional divers. Before the experiment, all volunteers declared in a questionnaire that they did not smoke or drink alcohol. Furthermore, 72 hours prior to the experiment the volunteers did not do any excessive physical activity. Anthropometric parameters for the study group are shown in Table 1.

#### 2.1.1. Hyperbaric Exposure

Hyperbaric exposures were performed at the Department of Diving Equipment and Technologies for Underwater Works, Polish Naval Academy in Gdynia, at the hyperbaric chambers complex DKN-120. The exposures were performed by qualified personnel. Before and after each exposure the subject underwent a full medical examination. The use of the hyperbaric chamber allowed creating comparable exposure conditions to be created for all subjects. The expositions in hyperbaric chamber were performed in three-person groups. Factors such as a breathing gas used, physical exercise, ambient temperature, and humidity were monitored in all experiments and then were registered throughout the exposure. All subjects used air as a breathing gas and were not exposed to physical exercise. The exposure conditions imitated pressure conditions during diving to 30 meters of sea water.

**Figure 1:**
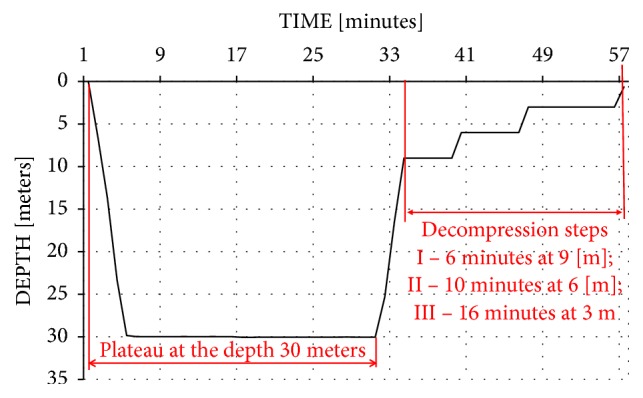
The depths and durations of the decompression stops.

The volunteers were placed in a hyperbaric chamber and exposed to compression of 40 MPa (total pressure was 0.4 MPa : 0.3 MPa in the chamber + 0.1 MPa of the atmospheric pressure). The exposure plateau was equal to ca. 30 minutes, followed by gradual decompression, according to the decompression tables of the Polish Navy (Figure 1). For safety reasons and in order for the side effects to be minimalised, e.g., “bends” after exposure to 30 meters, the decompression pattern used resembled that followed after diving to 33 meters.

#### 2.1.2. Biochemical Parameters

Blood collected in the antecubital vein was used for biochemical analyses. Samples were collected in tubes without anticoagulant (approx 3 mL) to obtain serum for BNP, ANP, angiotensin, angiotensin (1–7), and urotensin II determinations (Vacuette ®Greiner Bio-One). Plasma obtained from blood collected from the same vein on anticoagulant, being 2 ml of EDTA, was used to determine L-arginine levels. Then serum was separated by centrifuging at 2500 ×g at +4°C for 15 minutes. Serum and plasma were frozen at −80°C and stored until used for determinations.

All parameters were determined using commercially available ELISA kits (Cloud-Clone Corp, USA), in accordance with the instruction provided with each kit.

#### 2.1.3. Cardiovascular and Autonomic Parameters

Before, and immediately after, the exposure the volunteers underwent an examination with the Task Force Monitor (TFM, CNSystems, Medizintechnik, Graz, Austria). The TFM performs automated and computed beat-to-beat analysis of the heart rate, electrocardiogram (ECG), impedance cardiography (ICG), oscillometric and noninvasive continuous blood pressure measurements (oscBP, contBP). The calculation of hemodynamic and autonomic parameters is based on the above-mentioned biological signals. Furthermore, the TFM enables continuous (beat-to-beat), reliable, and reproducible measurements of all the parameters [29–31]. The TFM device enables determination of many basic and advanced cardiovascular parameters, including fluid changes and peripheral resistance. The autonomic nervous activity is also derived from heart rate variability (HRV) and blood pressure variability (BPV) by means of spectral analysis, and spontaneous baroreceptor sensitivity (BRS) is analyzed with the sequential method. In addition, basic statistics of all parameters were calculated automatically for defined periods

#### 2.1.4. Statistics

All data are presented as means ± SD. Normal distribution of the study variables was verified with the Shapiro-Wilk test. Levene's test was used to check the homogeneity of variances in the analyzed samples. Depending on distribution characteristics of analyzed variables, the independent samples Student *t*-test or Wilcoxon's-pairs test was used to evaluate significance of differences between measured values. All calculations were performed with the package Statistica 10 (StatSoft), with the assumed level of statistical significance of *α* < 0.05.

## 3. Results

### 3.1. Biochemical Analysis

When peptide measurements taken before and after hyperbaric exposure were compared among the 18 participants, a statistically significant increase was seen in both atrial ANP and ventricular BNP. For L-Arg and urotensin II levels, a significant reduction in the level of this amino acid was observed following exposure in the hyperbaric chamber (Table 2). Percentage indicators of the increase and decrease of the biochemical parameters values were shown in Table 2.

### 3.2. Cardiovascular Assessment

**Figure 2:**
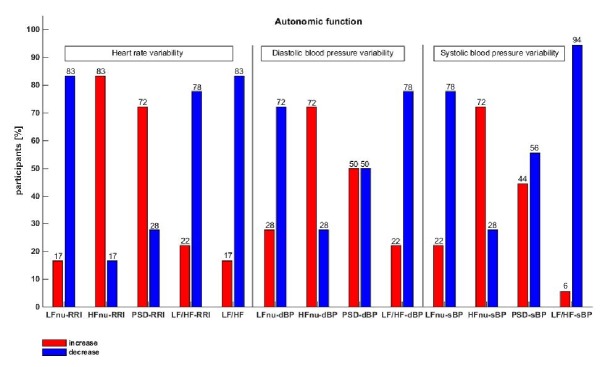
Subjects percentage indicators of the increase and decrease of the autonomic parameters: LFnu-RRI, HFnu-RRI, PSD-RRI, LF/HF-RRI, LF/HF, LFnu-dBP, HFnu-dBP, PSD-dBP, LF/HF-dBP, LFnu-sBP, HFnu-sBP, PSD-sBP, and LF/HF-sBP.

When cardiovascular and autonomic parameters were measured at rest in the 18 participants there were significant reductions in heart rate and cardiac index and significant increases in diastolic and mean arterial blood pressure with a parallel increase in total peripheral resistance after hyperbaric exposure. In addition left ventricle work index parameter significantly changed with reductions in left ventricular work index and ejection rate and increases in left ventricular ejection time and preejection time. Data in Figure 2 and Table 3 presented percentage indicators of the increase and decrease of the cardiovascular function.

### 3.3. Autonomic and Baroreceptors Parameters

When we explored changes in heart rate and blood pressure variability on hyperbaric exposure, we showed a significant reduction in low frequency parameters (largely sympathetic) and increase in high frequency (largely parasympathetic) with an associated shift in sympathovagal balance after hyperbaric exposure. The LF/HF ratios calculated from all bands of heart rate variability and blood pressure variability were elevated in HF component, and thus sympathovagal balance was decreased in response to hyperbaric exposure or/and hyperoxia or a combination of both.

There were no significant changes in baroreceptors reflex sensitivity in all sequences, as significant decrease was observed in numbers of detected baroreceptor measures.  Table 4 shows percentage indicators of the increase and decrease of the autonomic function indicators.

## 4. Discussion

This study has confirmed that exposure to a hyperbaric environment has a significant impact on the cardiovascular system. This is confirmed both by changes in peptides associated with poorer cardiovascular outcomes, where a significant increase in the studied parameters was observed, and by noninvasive examination with the TFM system.

In the experiment, the influence of other factors, such as physical activity or stress associated with being in water (hypothermia, energy needed for sustaining physical activity which is connected with the increased oxygen consumption), was minimised. This is of particular importance as previous studies have reported similar changes as those seen in the current study, but changes have been interpreted as being the effect of stress caused by being underwater, the ambient temperature, and the physical activity of the divers [32–34]. Despite intense studies conducted in recent years, the authors have not found a publication presenting such results concerning the direct effect of external pressure on the cardiovascular system.

Previous studies have confirmed an increase in the level of type B natriuretic peptide observed after diving [35–37]. It is important to note that a significant increase in BNP or proBNP after exposure to HBO observed in these papers was interpreted as occurring because of physical exercise; however, in our experiment this factor was eliminated, and still we observed a statistically significant increase that was within a population reference range for that factor. Our results support the following mechanism, typical also for water immersion: exposure to pressure in hyperbaric chambers results in an increase in intrathoracic blood volume, causing dieresis because of a release of natriuretic hormones and suppression of antidiuretic hormone [38–40].

We also found that the hyperbaric environment causes an L-Arg change, which has influence on nitric oxide (NO) production in vessel endothelium. Endothelium derived nitric oxide seems to be released as free NO radical or in a more stable form (interactions with thiol or non-haem iron ligands). Under resting conditions of flow, NO provides a constant vasodilator tone acting against sympathetic vasoconstriction. Following changes in environment, increased shear stress during blood shifts, NO synthesis can be stimulated to provide a mechanism for the local regulation of vascular tone and blood flow [41, 42]. Observation showing the absence of microvascular vasoconstriction during hyperbaric hyperoxic conditions also supports our interpretation [43].

It is worth mentioning that we also observed a decrease in urotensin II secretion in the hyperbaric environment. Urotensin II is considered to be the most effective vasoconstrictor, and it appears that it can be manipulated in the hyperbaric environment. There seems to be a balance between endothelium-independent vasoconstriction and endothelium-dependent vasodilatation (urotensin II/NO) [44].

Our study has confirmed that exposure to hyperbaric pressure has no significant effect on angiotensin (Ang) but we did find a decrease in Ang-(1–7). It is well known that nitric oxide (NO) release is promoted by Ang-(1–7) through activation of endothelial NO synthase (eNOS) and neuronal NO synthase. A few studies have shown a significant vasodilator effect of Ang-(1–7) in isolated cardiomyocytes. Great part of the research concerning Ang-(1–7) or other agonists in the heart puts strong emphasis on its cardioprotective effects [45].

A significant reduction of ischemia/reperfusion is produced mainly by low concentrations of Ang-(1–7). Even though Ang-(1–7) is formed, it is observed that the blood vessels are of the major areas where the above mentioned low concentrations occur. Vasodilation in aortic rings and in several vascular territories is produced by Ang-(1–7), the effect of which leads to a decrease in total peripheral resistance with a consequent increase in cardiac output.

These essentially equivalent alterations have hemodynamic effect on blood pressure leading to no net change in blood pressure. A right ventricular overload being an impairment of ventricular diastolic performance may be observed as an indication of the fact that circulating gas bubbles during decompression are associated with cardiac changes. There is a hypothesis of “silent” gas bubbles damaging pulmonary endothelium and activating the reactive systems of the human body with which the changes in biochemical parameters are consistent [38, 39, 46–48]. The results shown in our biochemical studies do not exceed reference ranges; however, a statistically significant increase indicates the hyperbaric environment is not without potential impact upon the human body. A decrease in HR, an increase in mBP, dBP, and TPR, and increase in parasympathetic heart nerves activity suggest an increase in heart afterload with a decrease in heart activity (HR and contractility) almost one hour after hyperbaric exposure. These prolonged effects seem to be related to resetting of different cardiovascular reflex mechanisms, activated by mechanical stimulus, hyperbaric pressure, resulting in central blood shifts and higher levels in parameters concerning central venous pressure (increase in heart preload). Changes of sympathoparasympathetic balance of heart innervation with increased vascular resistance (increase in sympathetic vascular drive) indicate changes of regulatory pattern in cardiovascular system. This difficult (hard) haemodynamic situation helps to explain cardiac disorders and an unexpected decease during diving activities. It is noticed that divers whose age ranges from 60 to 70 years are highly probable to suffer from diving-related deaths resulting from cardiovascular disease [12, 49–51]. The decrease in respiratory cardiac arrhythmia in subjects from this age-group indicates the decrease in tonic cardiac parasympathetic activity. Bradycardia found in our study might be considered as a protective effect on cardiac muscle in hyperbaric condition. Our result confirms importance of being properly examined by a medical practitioner before diving. The requirements for that activity differ depending on the purpose of diving itself; for military divers they are more stringent than for recreational divers. Hyperbaric chamber workers, caisson, and tunnel workers all should have unique standards. The crucial element in those requirements, especially in the sport and work diving community, is a thorough verification of diving candidates for the presence of a coronary disease. Those with an increased risk for coronary disease due to age and chronic illness (metabolic) should not be selected for this type of activity. We would conclude that further studies are required to explore whether in long-term professional divers, who are undergoing deep hyperbaric exposures, experience delayed effects particularly related to cardiovascular diseases, such as LVH.

## Figures and Tables

**Table 1:** Demographics of the study group.

Characteristic	Hyperbaric exposure *n* = 18
Age [years]	31.1 ± 5.9
Body mass [kg]	82.9 ± 13.4
Body height [m]	1.79 ± 0.08
Body Mass Index [kg/m^2^]	25.6 ± 2.7
Heart Rate at rest [*n*/min]	70.2 ± 9.1
sBP at rest [mmHg]	118.8 ± 12
dBP at rest [mmHg]	72.6 ± 6.8
mBP at rest [mmHg]	91 ± 8.2
diving experience [years]	8.7 ± 4.3

**Table 2:** Biochemical analysis results before and after hyperbaric exposure. The +/− mean values of standard deviation were presented.

Parameter	Hyperbaric exposure *n* = 18 subjects		
	before_exposure	after_exposure	*P*
L-Arginine (ARG) [*μ*g/mL]	87.1 ± 17.7	75.4 ± 15.3	**<0.01**
Brain natriuretic peptide (BNP) [pg/mL]	33 ± 21.9	46.5 ± 27.1	**<0.01**
Atrial natriuretic peptide (ANP) [pg/mL]	37.8 ± 24	47.2 ± 26.6	
Angiotensin (ANG) [pg/mL]	4.7 ± 0.7	4.4 ± 0.6	0.1752
Angiotensin (1–7) (ANG 1–7) [ng/mL]	327 ± 117.1	266.7 ± 98.8	**<0.01**
Urotensin II (URO_II) [ng/ml]	119.6 ± 47.6	91.2 ± 35.5	**<0.01**

**Table 3:** Cardiovascular function before/after hyperbaric exposure. The +/− mean values of standard deviation were presented.

Parameter	Hyperbaric exposure *n* = 18 subjects		
	before_exposure	after_exposure	*P*
HR [*n*/1]	70.2 ± 9.1	60.7 ± 7.2	**0.0002**
sBP [mmHg]	118.8 ± 12	123.6 ± 10.3	0.0526
dBP [mmHg]	72.6 ± 6.8	77.8 ± 7.2	**0.0013**
mBP [mmHg]	91 ± 8.2	96 ± 8	**0.0032**
SI [ml/m^2^]	54.4 ± 11.1	53.8 ± 9.9	0.5565
CI [l/min/m^2^]	3.8 ± 0.9	3.2 ± 0.6	**0.0018**
TPRI [dyn*∗*s*∗*m^2^/cm^5^]	1995.8 ± 667	2429.2 ± 681.9	**0.0028**
IC [1000/s]	63 ± 18.2	60.3 ± 16.4	0.2310
ACI [100/s^2^]	85.3 ± 28.8	79.1 ± 25.7	0.1226
HI [1/s^2^]	0.4 ± 0.1	0.3 ± 0.1	0.1329
LVWI [mmHg*∗*l/[min*∗*m^2^]]	4.6 ± 1.2	4.1 ± 0.8	**0.0477**
LVET [ms]	312.6 ± 12.5	323.3 ± 15.1	**0.0015**
PEP [ms]	105.4 ± 12.9	109.6 ± 10.5	**0.0385**
ER [%]	36.4 ± 3.7	32.5 ± 2.7	**0.0002**

**Table 4:** Autonomic and baroreceptors function before/after hyperbaric exposure. The +/− mean values of standard deviation were presented.

Parameter		Hyperbaric exposure *n* = 18		
		before_exposure	after_exposure	*P*
Heart rate variability	LFnu-RRI [%]	62 ± 14.1	45.6 ± 20	**0.0013**
	HFnu-RRI [%]	38 ± 14.1	54.4 ± 20	**0.0013**
	PSD-RRI [ms2]	2218.5 ± 2387	2850.5 ± 2885.2	**0.0279**
	LF/HF-RRI [*n*/1]	2.2 ± 1.3	1.4 ± 1.4	**0.0123**
	LF/HF [*n*/1]	1.8 ± 0.8	1.1 ± 1.2	**0.0156**

Diastolic blood pressure variability	LFnu-dBP [%]	54.5 ± 12	40.7 ± 18.5	**0.0065**
	HFnu-dBP [%]	10.9 ± 7.1	19.9 ± 14.4	**0.0222**
	PSD-dBP [mmHg2]	9 ± 5.6	8.7 ± 7.1	0.4724
	LF/HF-dBP [*n*/1]	7.5 ± 4.6	3.7 ± 3	**0.0057**

Systolic blood pressure variability	LFnu-sBP [%]	48.6 ± 14.1	38.2 ± 19.5	**0.0222**
	HFnu-sBP [%]	15.8 ± 10.3	22.1 ± 12	**0.0123**
	PSD-sBP [mmHg2]	12.8 ± 9.9	11.4 ± 8.2	0.5861
	LF/HF-sBP [*n*/1]	4.9 ± 4	2.5 ± 1.9	**0.0006**

Baroreceptors reflex sensitivity	Up-Events [*n*/1]	12.8 ± 9.1	6 ± 5.5	**0.0008**
	Down-Events [*n*/1]	11.9 ± 9.3	5.1 ± 5.5	**0.0026**
	Total-Events [*n*/1]	24.8 ± 18.1	11.1 ± 10.7	**0.0065**
	Up-Events Slope [ms/mmHg]	27.1 ± 22	26.6 ± 13.3	0.3061
	Down-Events Slope [ms/mmHg]	16.2 ± 8.9	22.3 ± 11.5	0.0597
	Total-Events Slope [ms/mmHg]	22.6 ± 15.3	24.7 ± 10.8	0.2485

## Data Availability

The datasets generated and/or analyzed during the current study are available from the corresponding author upon request from other scientists.

## Abbreviations

ACI: Acceleration index
ANP: Atrial natriuretic peptide
BNP: Brain natriuretic peptide
BSA: Body surface area
CI: Cardiac index
contBP: Continuous blood pressure
CV: Cardiovascular
dBP: Diastolic blood pressure
ECG: Electrocardiogram
eNOS: Nitric oxide synthase 3
ER: Early ejection
HF: High frequency
HFnu: Normalised units high frequency
HI: Heather index
HR: Heart rate
IC: Index of contractility
ICG: Impedance cardiography
LF: Low frequency
LFnu: Normalised units low frequency
LVET: Left ventricular ejection time
LVWI: Left ventricular work index
mBP: Mean blood pressure
NO: Nitric oxide
oscBP: Oscillometric blood pressure
PEP: Aortic preejection period
PSD: Power spectral density
sBP: Systolic blood pressure
SI: Stroke index
TFM: Task Force Monitor
TPR: Total peripheral resistance
TPRI: Total peripheral resistance index
URO_II: Urotensin II.
